# EXPLORING THE NARRATIVE OF AGING THROUGH MUSIC

**DOI:** 10.1093/geroni/igac059.961

**Published:** 2022-12-20

**Authors:** Desmond O'Neill

**Affiliations:** Trinity College Dublin, Dublin, Dublin, Ireland

## Abstract

With increasing interest in exploring narratives of aging through literary/oral sources, a less prominent area of inquiry has been possibilities afforded by narratives inherent in music across the life-span and into later-life. Two possible areas of interest are the arc of development and change over the life-course, and inherent content of the music. The latter element is less prominent in narrative analysis, providing a potentially rich field of discovery. While popular song parallels these possibilities, further opportunities arise from instrumental, orchestral and operatic music, allied to strengths in musicological analysis. Later compositions whose elements provide insights into life course review (Shostakovich Viola Sonata and Metamorphosen, Richard Strauss) as well as romantic love in later life across generations (The Cunning Little Vixen, Leos Janáček). The analysis will seek to delineate to possible avenues of interpretation of musicological analysis in understanding the phenomenology and opportunities arising from aging.

